# Suppression of inflammatory genes expression in the injured host intestinal wall during *Mesocestoides vogae* tetrathyridium larvae migration

**DOI:** 10.1371/journal.pntd.0008685

**Published:** 2020-10-13

**Authors:** Kei Hayashi, Rinako Sugisawa, Taizo Saito, Toshiyasu Matsui, Yuji Taniguchi, Tatiana Batanova, Tokuma Yanai, Jun Matsumoto, Katsuya Kitoh, Yasuhiro Takashima

**Affiliations:** 1 Laboratory of Veterinary Parasitology, Faculty of Veterinary medicine, Okayama University of Science, Ikoinooka, Imabari, Japan; 2 Department of Veterinary Parasitology, Gifu University, Yanagido, Gifu, Japan; 3 The United Graduate School of Veterinary Science, Gifu University, Yanagido, Gifu, Japan; 4 Laboratory of Veterinary Anatomy, Faculty of Veterinary Medicine, Okayama University of Science, Imabari, Japan; 5 Laboratory of Veterinary Pathology, Gifu University, Yanagido, Gifu, Japan; 6 Laboratory of Medical Zoology, Department of Veterinary Medicine, Faculty of Bioresource Sciences, Nihon University, Kameino, Fujisawa, Japan; 7 Center for Highly Advanced Integration of Nano and Life Sciences, Gifu University, Yanagido, Gifu, Japan; Universidad de la Republica, Uruguay, URUGUAY

## Abstract

*Mesocestoides vogae* is a cestode parasite of the family Mesocestoididae (order Cyclophyllidea). Its larvae, tetrathyridium, are approximately 1 mm long and 300 μm wide and infect a wide range of host species including humans. Tetrathyridium migrate through the intestinal wall to invade the peritoneal cavity. Despite intestinal penetration by such a large-sized parasite, symptomatic intestinal disorders are not common during the migration period. In this study, the dynamics of tetrathyridia migration and their pathogenicity towards intestinal tissues were examined in mice infected orally with these parasites. Most tetrathyridia were found to migrate through the intestinal wall, moving into the peritoneal cavity or liver 24 to 48 hours after the oral infections. Next, the pathogenicity of tetrathyridium in the intestinal wall was histopathologically evaluated, and tissue injury from tetrathyridium migration was confirmed. Inflammatory foci were observed as tetrathyridium migration tracks from 48 hours after oral infection; however, the number of inflammatory foci had decreased by half more than 48 hours later. Therefore, we examined the gene expression levels of the macrophage driving cytokine, IL-1β, and the eosinophil recruiting chemokine, CCL11, by quantitative reverse-transcriptase PCR. The expression levels of these genes in the infected group were significantly lower than those of the non-infected group at 48 hours post-infection. Although the immunomodulating ability of the excretory-secretory products released from tetrathyridium has been previously shown by *in vitro* assays, the significance of this ability in their lifecycle has remained unclear. In this study, we discovered that tetrathyridium causes temporal inflammation in the intestinal wall during penetration and large-scale migration in this organ, but tetrathyridium simultaneously suppresses the host’s inflammatory gene expression, might to be a strategy that reduces inflammatory responses and increases survival of the parasite.

## Author summary

Excretory-secretory (ES) products are released by parasitic helminths into their migration sites and/or the intestinal regions they inhabit where parasite and host immune responses interact. ES products are release by a wide range of parasitic helminths, some of which are known to modulate the host’s immune system. Some ES products from some cestode parasites are known to reduce the production of pro-inflammatory cytokines from artificially stimulated cells under *in vitro* conditions. However, the immunomodulatory properties of the ES products have only been observed with *in vitro* experimental models and the biological consequences of their potential ability to suppress the host’s immune system during the parasite’s lifecycle and how they affect host–parasite interactions await discovery. Our results show that tetrathyridium, the larval stage of the *Mesocestoides vogae* cestode, strongly inhibits the host’s inflammatory gene expression in the injured intestinal wall, and that the inflammatory hot-spots caused by larval migration disappear almost immediately after mice are orally infected with these parasites. The ability to suppress the host’s inflammatory gene expression when larvae migrate through and damage the host’s tissues is an effective survival strategy for *M*. *vogae* intestinal parasites.

## Introduction

*Mesocestoides vogae* is a cestode belonging to the family Mesocestoididae within the order Cyclophyllidea. This cestode requires two intermediate hosts, coprophagous mites and many species of vertebrates [1, 2], as well as carnivores, the definitive hosts [3, 4]. The larvae (referred to as tetrathyridium) in the second intermediate hosts are orally infective to the definitive host or to a wide range of paratenic hosts, mammals, birds, reptiles and amphibians. When tetrathyridium infects a definitive host, it differentiates to an adult worm in the intestinal tract [3, 4]. In contrast, when tetrathyridium infects paratenic host animals, it does not differentiate, but migrates through the intestinal wall to invade the peritoneal cavity. Next, the tetrathyridium-stage parasite reproduces asexually in the peritoneal cavity or invades the liver [5]. Proliferation at this site is known to cause peritoneal larval cestodiasis, a form of life-threatening peritonitis in mammalian hosts [6–10]. *M*. *vogae* has been studied as a model cestode, and the pathology it causes in the liver and its asexual reproduction in the peritoneal cavity of rodent hosts has been well investigated [7, 11–13]. Nevertheless, studies on its pathogenicity in the intestinal tissues and the acute clinical manifestations that occur during its intestinal migration have been quite limited. Regardless of the large size of the tetrathyridium-stage parasite, which is approximately 1 mm long and 300 μm wide, intestinal perforation during tetrathyridium migration and obvious intestinal disorders are not common during this activity. The mechanism involved in asymptomatic perforation, when it does occur, is not yet known.

Excretory-secretory (ES) products are released from parasitic helminths into host-parasite interactive environments [14]. Components of the ES products vary by the parasite species and by the developmental stages from which they arise [14–16]. Interestingly, based on *in vitro* analyses, some ES product components have been characterized as immunomodulators. The ES products from *Spirometra erinaceieuropaei* plerocercoid cestodes reportedly suppress the gene expression of pro-inflammatory cytokines in lipopolysaccharide (LPS)-stimulated macrophages [17–20]. ES products from the *Taenia crassiceps* tapeworm suppress the proliferation response and cytokine production in mitogen-stimulated CD4+ enriched splenocytes [21]. It has also been reported that inflammatory cytokine production in cultured bone marrow-derived dendritic cells can be reduced by impregnating them with the ES product from *M*. *vogae* in the culture medium [22, 23]. However, such immunomodulation by cestode ES products was only observed using *in vitro* experimental models and the function and/or impact of such immunosuppressive abilities in their lifecycles or on host-parasite interactions remains elusive. In this study, the dynamics of tetrathyridium migration in orally-infected mice was investigated, and inflammatory response processes and cytokine expression in the injured mouse intestinal wall were monitored.

## Methods

### Ethics statement

This study was carried out in strict accordance with the recommendations in the Guide for the Care and Use of Laboratory Animals of the Gifu University. The protocol was approved by the Committee on the Ethics of Animal Experiments of Gifu University (16007). Collection of parasites and/or tissue samples from mice were performed after the euthanasia by cervical dislocation. Oral administration of parasites and euthanesia by cervical dislocation were carried out only by skilled researchers.

### Mice and parasites

Female Balb/c mice of 8–9 weeks of age (Charles River Laboratories Japan Ins., Yokohama, Japan) were used for the investigations and for passaging *M*. *vogae* tetrathyridium. Transgenic mice expressing green fluorescent protein (GFP), C57BL/6-Tg (CAG-EGFP) C14-Y01-FM131 Osb (GFP^+/+^ mice) [24, 25] were used for the confocal laser microscopic observations. *M*. *vogae* tetrathyridium were maintained in Balb/c mice via oral infection and harvesting from their peritoneal cavities at least 60 days after infection commencement. The harvested tetrathyridium parasites were washed with phosphate-buffered saline (PBS) three times and then maintained in RPMI 1640 medium (Sigma Aldrich, St. Louis, MO) containing 20% fetal calf serum (FCS), 75 U/ml penicillin and streptomycin, and were incubated for up to seven days at 37°C in a 5% CO₂ incubator before next the infection.

### Tracking tetrathyridium migration in mice

Three hundred tetrathyridia were used to orally infect Balb/c mice, and the whole intestines and liver were collected from each mouse at 24 or 48 h post-infection. The intestines were cut in cross section every 1 cm, and each part was opened up to form a sheet. The number of tetrathyridia on the liver surface, sticking to the intestinal wall, or remaining in the abdominal cavity were counted under a stereo microscope. Tetrathyridium sticking to the intestinal wall were observed from both inside and outside of the intestinal sections. Three mice were used for each group and the experiments were repeated twice independently.

### Fluorescent staining of live tetrathyridia

Tetrathyridia were stained with 40 μM CellTracker Red CMTPX Dye (Thermo Fisher Scientific, Tokyo, Japan) in RPMI 1640 medium containing 20% FCS, 75 U/ml penicillin and streptomycin, with incubation for 45 min at 37°C in a 5% CO₂ incubator. After incubation, the tetrathyridia were washed 3 times in RPMI 1640 medium containing 20% FCS, 75 U/ml penicillin and streptomycin, before setting up the infections. The red fluorescently stained live tetrathyridia used to orally infect GFP+/+ mice were used for the confocal laser microscopic observations.

### Tissue transparency

Five hundred of the red fluorescently stained live tetrathyridia were used to orally infect GFP^+/+^ mice, and the small intestines from the pyloric region and 8 cm beyond it were collected from each mouse at 24 h post-infection. The intestines were cut into 2-cm cross sections and each section was opened up to form a sheet, and then pre-fixed in 4% paraformaldehyde overnight. The fixed intestinal sections were made transparent using a previously established method [26]. Briefly, the fixed intestines were sliced into 1 mm thicknesses and soaked in 20% fructose with α-thioglycerol (fructose solution) with shaking at 20 rpm for 4 h. The same operations were carried out with 40% and 60% fructose solution for 4 hours each, and with 80% and 100% fructose solution for 12 h each. After that, the samples were soaked in 115% fructose solution (SeeDB solution) followed by shaking at 10 rpm for 24 h in the dark. The transparent tissues were observed under a confocal laser microscope.

### Transmission electron microscopic observations

Balb/c mice were orally injected with five hundred tetrathyridia or PBS, and small intestine from each mouse was collected at 24 h post-infection. The intestinal sections (1- to 3-mm square) confirmed to contain tetrathyridium using a stereomicroscope were excised and pre-fixed in 0.1 M phosphate buffer containing 2% glutaraldehyde and 2% paraformaldehyde. Post-fixation was carried out for 2 h with 2% osmium tetroxide aqueous solution at 4°C. Dehydration was carried out with 50% to 100% ethanol (15 min each) at 4°C to room temperature, and the samples were then embedded by epoxy thermal polymerization (EPON 812, Shell Chemicals, USA) for 48 h at 60°C. The samples were sliced into 80 to 90 nm thicknesses using an ultramicrotome, subjected to double staining electron dyes using uranyl acetate and lead dye solution, and then observed with an H-7600 transmission electron microscope (Hitachi, Tokyo, Japan).

### Histological analyses

Six hundred tetrathyridia were used to orally infect Balb/c mice, and 1 to 5 cm of the small intestine from the pyloric region onwards was collected from each mouse at 48 and 96 h post-infection. Each intestine was divided into 16 sections by cutting every 2.5 mm in cross section, and then fixed in 4% paraformaldehyde overnight. The fixed intestines were sectioned so that the cross sections could be seen, stained with hematoxylin-eosin solution (HE), and then observed under a bright-field optical microscope. Regions where more than 30 inflammatory cells were recognized were defined as “inflammatory areas”, and the number of inflammatory areas was counted and degrees of seriousness of each the inflammation were scored for all the 16 sections. The score was calculated based on two factors; damaged tissue area, and size of inflammatory area. Damaged tissue areas were evaluated as follow with score ranging 1 to 4, 1: one area of lamina propria, submucosa, circular layer and longitudinal layer, 2: two of the four area, three of the four area, and all of the four area was damaged. Size of inflammatory area measured with OLYMPUS cellSens Standard system version 1.18 (Olympus, Tokyo, Japan) and evaluated as follow with score ranging 1 to 4, 1: smaller than 0.01mm^2^, 2: smaller than 0.03mm^2^, 3: smaller than 0.1mm^2^, 4: larger than 0.1mm^2^. Three mice were used for each group and the experiments were repeated twice independently. The analyses were performed under double blind inspection.

### Immunohistochemistry

A macrophage specific protein Ionized calcium-binding adapter molecule 1 (Iba1) were immune-stained with the sectioned intestines which were same series with the histological analyses [27]. Tissue sections after deparaffinization were pretreated for microwave antigen retrieval in 0.01 M citrate buffer (pH 6.0) using a microwave oven. Sections were blocked with TSA blocking reagent (PerkinElmer, Waltham, MA, USA) and then incubated with goat anti-Iba1 antibody (1:500; ab5076; Abcam, Cambridge, UK) overnight at 4°C. The primary antibody was detected with biotinylated horse anti-goat IgG (1:400; Vector Laboratories, Burlingame, CA, USA) followed by Vectastain ABC-Elite kit (Vector Laboratories) and diaminobenzidine. Sections were counterstained with hematoxylin, dehydrated, and mounted with DPX mountant (Sigma-Aldrich, St. Louis, MO, USA) for microscope observation.

### PrimerArray analysis

Three hundred tetrathyridia were used to orally infect Balb/c mice, and small intestinal tissue 2 to 3 cm from the pylorus (approximately 50μg) of each mouse was collected 48 h post-infection. Total RNA was extracted from the tissues by using an RNeasy Mini kit (QIAGEN, Hilden, Germany) according to the manufacturer’s instructions. cDNA was synthesized from 500 ng of the RNA as the template using PrimeScript RT reagent kit (Perfect RealTime) (TaKaRa, Kusatsu, Japan). The cDNA quantities were examined with a kit designed to analyze the expression levels of 88 cytokine-related genes (PrimerArray Cytokine-cytokine receptor interaction; Mouse), according to the manufacturer’s instructions. The expression levels of these 88 genes were normalized based on the normalization factors which was calculated by using the expression levels of 8 housekeeping genes; glyceraldehyde-3-phosphate dehydrogenase (GAPDH), beta-actin (ACTB), phosphoglycerate kinase 1 (PGK1), peptidylprolyl isomerase A (PPIA), beta-2 microglobulin (B2M), glucuronidase, beta (GUSB), hypoxanthine guanine phosphoribosyl transferase 1 (HPRT1) and TATA box binding protein (TBP), using the application software PrimerArray Analysis Tool Ver.2.2 (TaKaRa). The experiments were repeated twice independently; once in n = 1 and once in n = 3, respectively. In the first experiment, the relative expression level of the 88 genes were compared with those of the naïve counterpart. In the second experiment, the relative expression level of the 88 genes in each infected individual were compared with those of the three naïve mice.

### Quantitative RT-PCR

The intestinal tissues from mice orally infected with tetrathyridia were collected using the same methods that were used for the PrimerArray analysis. Total RNA was extracted using TRIZOL Reagent (Thermo Fisher Scientific, USA) according to the manufacturer’s instructions. cDNA was synthesized from 500 ng of the purified RNA as the template using ReverTra Ace qPCR RT Master Mix with gDNA Remover (TOYOBO, Osaka, Japan) according to the manufacturer’s instructions. The real-time RT-PCR solutions contained 100ng of template cDNA, 0.4μM of each primer, 12.5μl of SYBR Premix Ex Taq II (Tli RNaseH Plus, TaKaRa), and distilled water in a final volume of 25 μl. The following primer sets were used: MA025939-F (5′-TCC AGG ATG AGG ACA TGA GCA C-3′) and MA025939-R (5′-GAA CGT CAC ACA CCA GCA GGT TA-3′) for interleukin 1β (IL-1β), MA030052-F (5′-CAG ATG CAC CCT GAA AGC CAT A-3′) and MA030052-R (5′-TGC TTT GTG GCA TCC TGG AC-3′) for C-C motif chemokine 11 (CCL11) (TaKaRa), and MA050371-F (5′-TGT GTC CGT CGT GGA TCT GA-3′) and MA050371-R (5′-TTG CTG TTG AAG TCG CAG GAG-3′) for glyceraldehyde-3-phosphate dehydrogenase (GAPDH). The thermal cycling conditions consisted of an initial denaturation at 95°C for 30 sec, followed by 40 cycles of 95°C for 5 sec and 60°C for 30 sec. The dissociation step consisted of one reaction at 95°C for 15 sec, 60°C for 30 sec and 95°C for 15 sec, which was added for the melting curve analysis. The Ct values of IL-1β and CCL11 were normalized using the Ct value of GAPDH as the internal control, and the relative gene expression values for IL-1β and CCL11 were calculated. This experiment was repeated three times independently, and the number of mice in each group in each experiment was 5, with the exception that one of the infected mouse groups contained only 4 mice at 48 hours post-infection. The analyses were repeated three times independently (in total, n = 14 or 15 in each group).

### Statistical analyses

Variances in the three repeated measurements from the quantitative RT-PCR results for the experimental groups were analyzed separately by ANOVA. Student *t*-test and Tukey-Kramer test, for two groups and multiple comparisons, respectively, were used to analyze the differences between each experimental group. Statistical significance was set at *p* < 0.05.

## Results

### Dynamics of tetrathyridium migration in the mouse digestive tract

**Fig 1:**
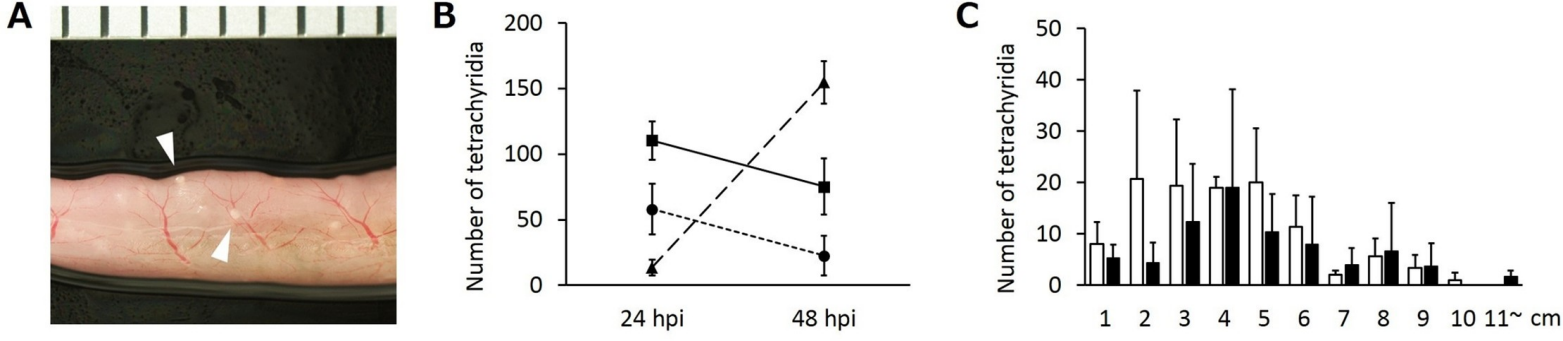
Dynamics of tetrathyridium migration in mice. One of the reproducible results of two repeated experiments is shown here. (A) Stereo-microscopic view of the small intestine showing penetrating tetrathyridium parasites 24 hours after the oral infection. The white arrowheads show the position of tetrathyridium parasites. One scale on the ruler represents 1 mm. (B) The number of tetrathyridia on the surface of the liver, sticking to the small intestinal wall, or remaining in the abdominal cavity 24 and 48 hours after the oral infection. Squares, circles and triangles represent the small intestine, the abdominal cavity, and the liver, respectively. Error bars: standard deviation. hpi; hours post-infection. (n = 3). (C) The number of tetrathyridia sticking to the small intestinal wall in each 1 cm area from the pylorus at 24 and 48 hours after the oral infection started. Intestinal areas 11cm from the pylorus are combined. White and black bars represent the number of tetrathyridia counted at 24 and 48 hours after the oral infection started, respectively. Error bars: standard deviation. (n = 3).

We first investigated the time scale and route of tetrathyridium migration in the orally infected mice. Tetrathyridia numbers in the liver, sticking to the intestinal wall or in the abdominal cavity of each mouse were counted 24 and 48 h post-oral infection. The independent experiments were repeated twice and reproducible results were obtained (Fig 1 and S1 Fig). Most tetrathyridia were observed sticking to the intestinal wall 24 h post infection, with few worms reaching the surface of the liver (Fig 1A and 1B and S1A Fig). Additionally, approximately one hundred tetrathyridia were observed on the inner gut lumen at this time point. However, most tetrathyridia were observed on the surface of the liver (Fig 1B and S1A Fig), with few observed on the inner gut lumen by 48 hours post-infection, showing that most of them had migrated through the intestinal walls from 24 to 48 h post oral infection. Furthermore, tetrathyridia sticking to the intestinal wall mainly 6 cm from the pyloric region were observed, and almost no perforation was evident 11 cm from the pylorus (Fig 1C and S1B Fig).

### Intestinal tissue injuries in the orally infected mice

**Fig 2:**
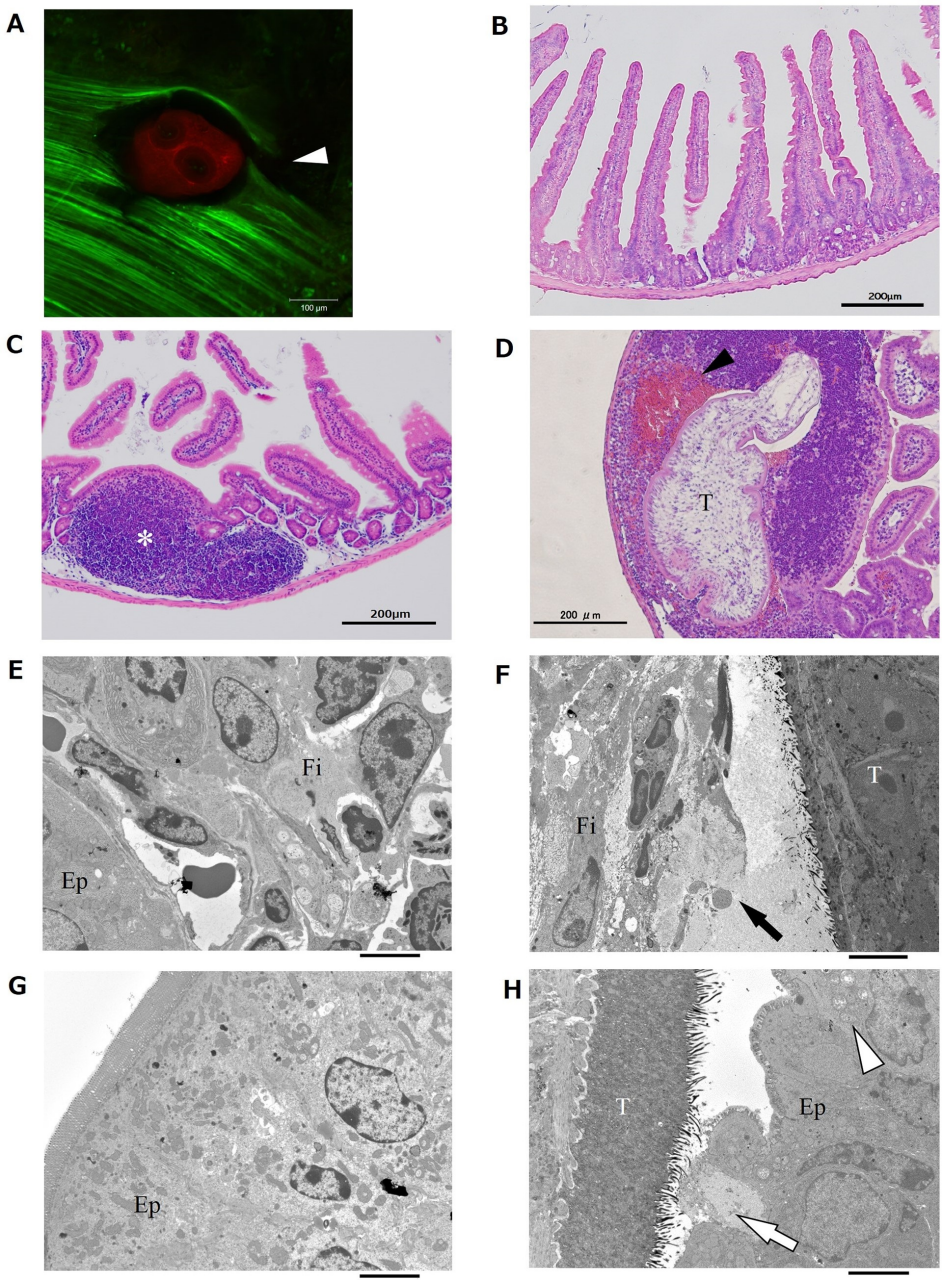
Tetrathyridium penetration and small intestinal wall tissue injury. One of the reproducible results of two repeated experiments is shown. (A) Confocal laser microscopic observation of the intestinal muscle layer penetrated by tetrathyridium. Green represents the muscle layer and red represents a tetrathyridium parasite. The white arrowhead indicates myofiber rupture. Scale bar, 100 μm. (B-D) HE stained histology of the small intestine 24 hours after the oral injection with PBS (B-C) or 500 tetrachyridia (D) started. T: tetrathyridium, white asterisk: lymph node, black arrowhead: haemorrhagia. Scale bar, 200 μm. (E-H) Transmission electron microscopic observations of the lamina propria (E-F) and villus (G-H) of the small intestine 24 hours after the oral injection with PBS (E, G) or 500 tetrachyridia (F, H). T: tetrathyridium, Fi: host fibroblast, Ep: host epithelial cell, black arrow: tissue injury in the lamina propria, white arrow: epithelial cell injury, white arrowhead: mitochondrial swelling. Scale bars, 4 μm.

Intestinal tissue injury as well as the migratory route taken by tetrathyridia was evaluated 24 hours after commencement of the oral infections. Confocal laser microscopic observations showed a partial loss of continuity of the muscular layers of the intestine, suggesting that the muscle layer was torn by tetrathyridium migration (Fig 2A). Our histological observations based on HE staining revealed the presence of large numbers of erythrocytes outside the blood vessels, suggesting that tetrathyridium parasites had ruptured the blood vessels (Fig 2B–2D). Transmission electron microscopic observations revealed destruction of the cells in microvilli and lamina propria. Fibroblasts in lamina propria (Fig 2E and 2F) and epithelial cell in microvilli (Fig 2G and 2H) neighboring penetrating tetrachyridium were severely destructed. These results suggest that the migration procedure used by tetrathyridium inflicts heavy injuries on the intestinal tissues by destructing microvilli, lamina propria, the muscle layer and blood vessels cells. Indeed, regardless of the physical destruction of the intestinal tissue, no notable clinical signs, such as an aversion to spontaneous movement and licking of the abdomen and squashing of the lower abdomen against the floor which are typical sign of a visceral pain, were apparently observed in any groups during the experiment.

### Cellular responses in the intestine

**Fig 3:**
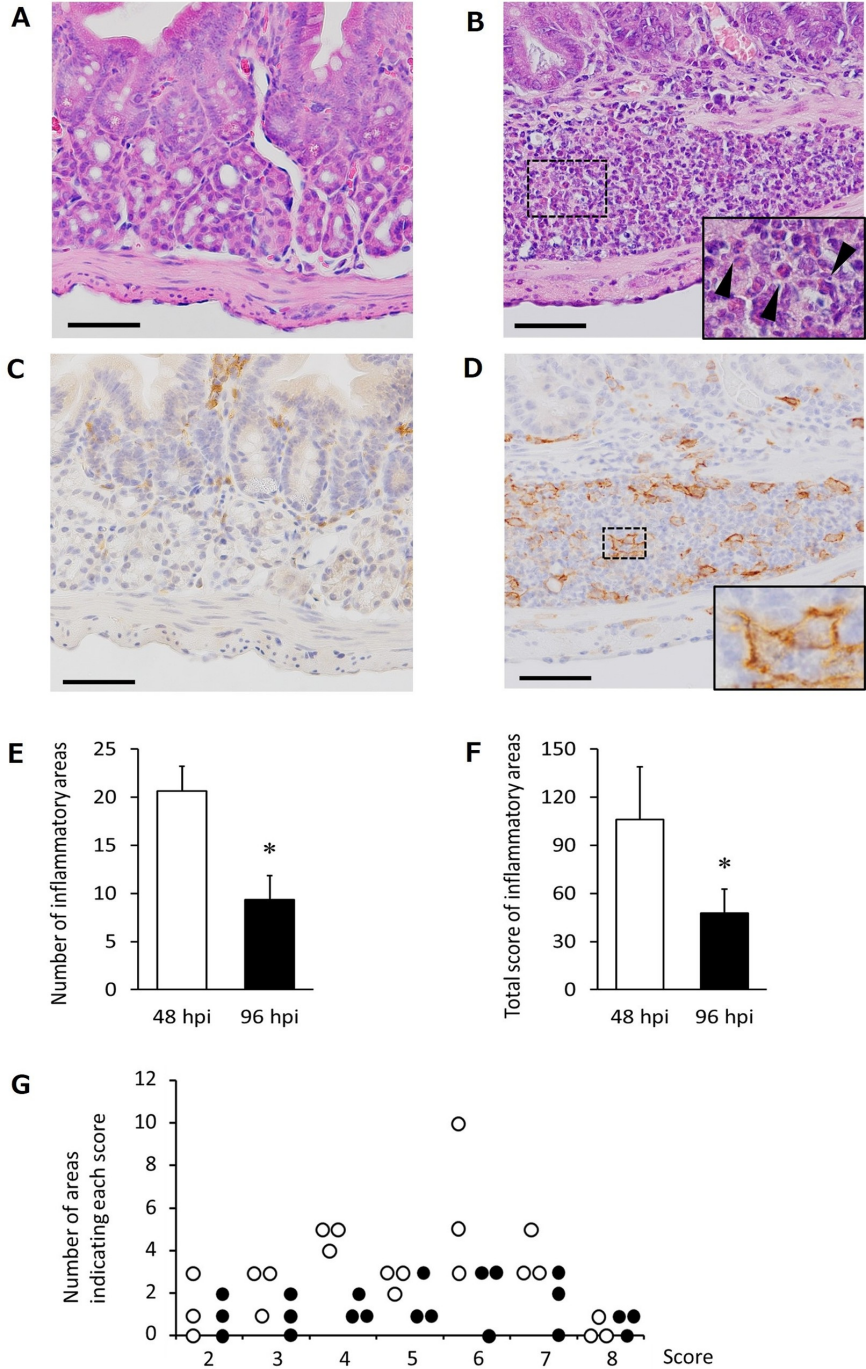
Disappearance of inflammatory responses. One of the reproducible results of two repeated experiments is shown. (A-B) HE stained histology of the mouse intestinal wall 48 h after oral injection with PBS (A) and 600 tetrathyridia (B). The bottom right panel is an enlarged view of the dashed square area. The arrowheads represent eosinophils. Scale bar, 100 μm. (C-D) Immune-staining with Iba1 (macrophage specific marker) of the mouse intestinal wall 48 h after oral injection with PBS (C) and 600 tetrathyridia (D). The bottom right panel is an enlarged view of the dashed square area. Scale bar, 100 μm. (E-F) Number (E) and total score (F) of inflammatory sites in the mouse intestinal wall at 48 and 96 h after oral infection. Error bars: standard deviation, *p<0.05. (n = 3). (G) The scores distributions of each inflammatory sites in the mouse intestinal tissues. White and black dots represent each individual at 48 and 96 h post infection groups, respectively. (n = 3).

Histopathological observations were conducted on the small intestine 48 and 96 hours after infection to investigate the cellular response induced by the tissue injury. Heavy inflammatory foci comprising macrophages and eosinophils were observed at the sites considered to be the tracks from tetrathyridia-stage penetration 48 hours after infection (Fig 3A–3D). However, in the two repeated experiments, the number of inflammation sites and total score of inflammatory areas decreased significantly after 96 h from infection commencement compared with that at 48 hours (Figs 3E, 3F, S2H and S2I), suggesting that the inflammation induced by tetrathyridium-stage migration was disappearing at 96 h post infection. The distributions of score of each inflammatory site were compared between the 48 h and 96 h post infection groups. As shown in Figs 3G and S2J, the frequency of inflammatory sites showing more than score 4 is lower in 96 h group.

### Relative expression levels of inflammatory cytokines

**Fig 4:**
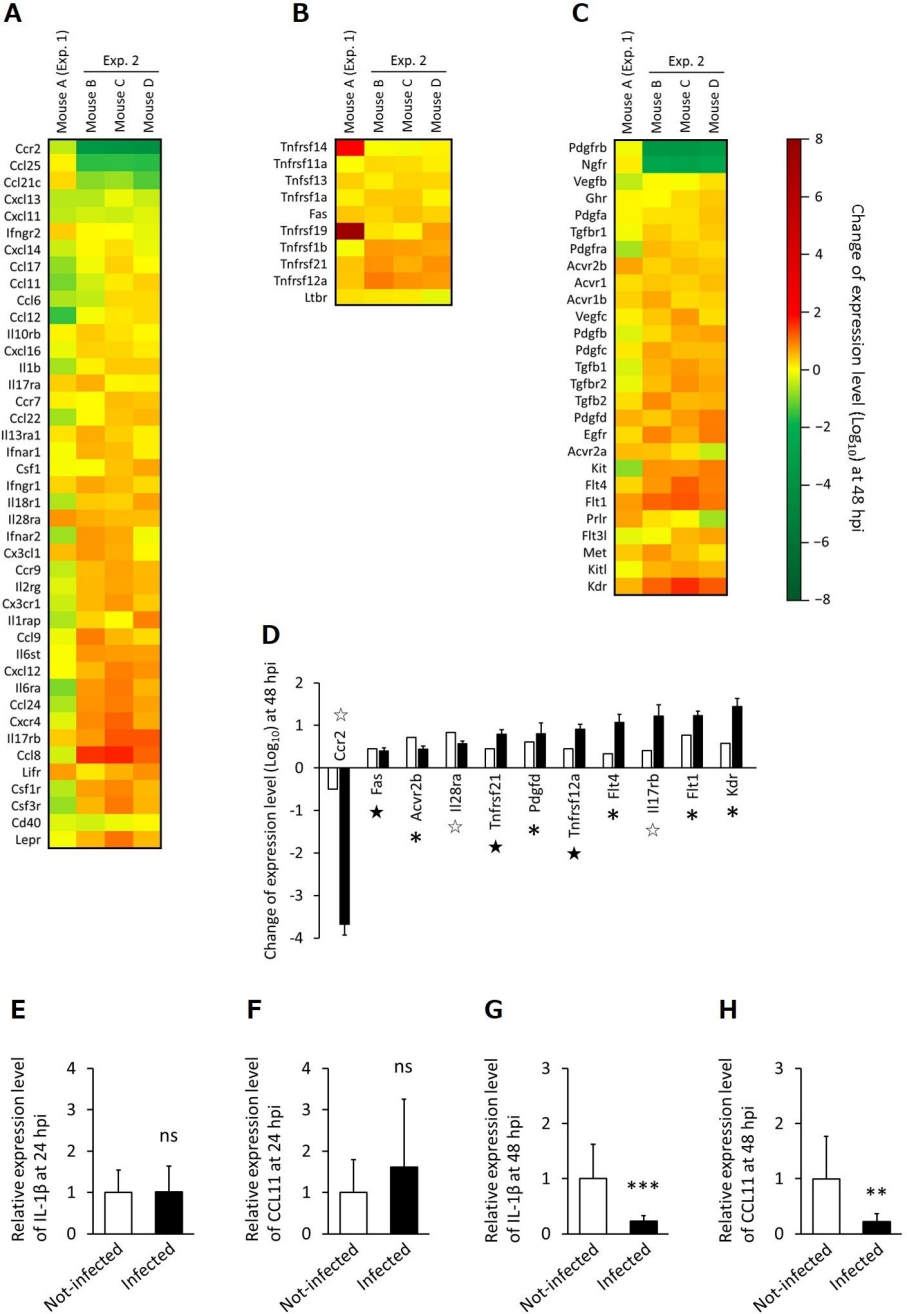
Relative gene expression levels of inflammatory response-related genes. (A-C) Heatmaps of relative expression levels of 79 inflammatory response-related genes; cytokine related genes (A), tumor necrosis factor related genes (B) and growth factor related genes (C) at 48h after 300 tetrathyridia infection in exhaustive quantitative analysis. The expression levels of the genes were normalized based on the normalization factors which was calculated by using the expression levels of 8 housekeeping genes, GAPDH, ACTB, PGK1, PPIA, B2M, GUSB, HPRT1 and TBP. Relative gene expression levels are indicated in colors from dark green (Log_10_ value: −8) to dark red (Log_10_ value: 8) as shown in color explanatory bar. Mouse A (n = 1) and mouse B-D (n = 3) were analyzed in the first and second experiment, respectively. The values of each gene relative expression are shown in S1 Table. (D) Relative gene expression levels of 11 genes that showed 2-fold higher or lower in all of the four infected mouse individuals in the exhaustive quantitative analysis. White and black bars represent the first and second experiment, respectively. Error bars show the standard deviations within the second experiment. Asterisks, White stars and black stars represent genes relating to cytokine, tumor necrosis factor and growth factor, respectively. (E-H) Quantitative RT-PCR analyses of IL-1β and CCL11 genes between the naïve and infected mice. The relative expression levels of (E) IL-1β at 24 hours after infection, (F) CCL11 at 24 hours after infection, (G) IL-1β at 48 hours after infection and (H) CCL11 at 48 hours after infection. Error bars: standard deviation, ns: not significant, **p<0.01, ***p<0.001. (n = 14 or 15 in total).

The rapid disappearance of inflammation in the mouse intestines prompted us to examine the expression levels of inflammatory cytokine genes in the injured intestinal tissue. Hence, we compared the expression levels of 88 inflammation response-encoding genes in the small intestine between the infected and non-infected mouse intestinal tissues 48 hours after oral infection. The expression level of 79 genes were successfully evaluated in all mice individuals in both of the two experiments (Fig 4A–4C). Among the 79 genes, 10 genes were 2-fold higher in all of the four infected mouse individuals; two genes, IL-17RB and IL-28RA, are cytokine related genes, three genes, TNFRSF12A, TNFRSF21 and FAS, are tumor necrosis factor related genes and five genes, KDR, FLT1, FLT4, PDGFD and ACVR2B, are growth factor related genes, respectively. On the other hand, one monocyte driven gene, C-C chemokine receptor type 2 (CCR2), was 2-fold lower in all of the four infected individuals (Fig 4D).

The phenomenon that most of the inflammatory cytokines were not up-regulated by the infection in the exhaustive quantitative analysis inspired us to investigate detailed expression levels of inflammatory cytokines, which are generally up-regulated during the acute infectious phase, with a time course. Then, we performed a quantitative RT-PCR analysis on two representative genes, IL-1β and CCL11, which are upstream factor of macrophage driving [28, 29] and eosinophil recruiting [30–34], respectively. The IL-1β and CCL11 expression levels did not differ between the infected and non-infected groups at 24 hours post-infection (Fig 4E and 4F). In contrast, at 48 hours post infection, the expression levels of these cytokines in the infected group were significantly lower than those in the non-infected group (Fig 4G and 4H). These results indicate that the expression levels of several inflammatory cytokines were strongly down-regulated to a lower level than those of the non-infected mice at 48 hours post infection.

## Discussion

*M*. *vogae* parasites cause severe peritonitis in mammalian hosts via their asexual proliferation and migration into the liver [6–10]. The pathology caused by this parasite in the liver and peritoneal cavity has been extensively investigated using rodent models [7, 11–13]. In these studies, experimental animals were not orally infected but directly injected with tetrathyridia intraperitoneally. Therefore, the dynamism and mechanism of tetrathyridium migration from the intestinal lumen into the peritoneal cavity has not been properly characterized. In this study, we discovered that tetrathyridium migrates through the intestinal wall of the duodenum and anterior part of the jejunum, mainly around 24 to 48 hours after commencement of an oral infection (Figs 1A–1C, S1A and S1B). The migration inflicts heavy injuries on the intestinal tissues of Fibroblasts in lamina propria (Fig 2F), epithelial cell in microvilli (Fig 2H), muscle layers (Fig 2A) and blood vessels (Fig 2D).

When tissues and cells are physically destructed, various types of molecules associated with damage-associated molecular patterns (DAMPs), such as ATP, DNA and histone proteins, are released from the injured cells [35]. The DAMPs bind to toll-like receptors (TLR) on various types of surrounding cells, except erythrocytes, and promote pro-inflammatory cytokine expression via the myeloid differentiation primary response gene 88 (MyD88) pathway [35, 36]. IL-1β is one of the major, primary pro-inflammatory cytokines expressed through the TLR-MyD88 pathway. In consequence to activation of the pathway, transcription factors such as nuclear factor-κB (NF-κB) and activator protein-1 (AP-1) regulate transcription of IL-1β gene, and the transcription activates the production of a proIL-1β protein. ProIL-1β protein is secondly regulated via processing into mature IL-1β as an activated form by caspase-1, and is extracellularly secreted [37–39]. IL-1β binds to IL-1 receptors (IL-1Rs) on neutrophils, monocytes and macrophages and activates them. The activated immune cells promote tissue inflammation by releasing various inflammatory cytokines and chemokines [28]. In addition, the IL-1R signaling pathway also promotes the release of IL-1β itself from the cells; therefore, IL-1β plays a central role via positive feedback in the inflammatory response [40]. Furthermore, when blood vessels are injured, various inflammation-promoting cells and substances such as leucocytes, thrombin, and fibrinopeptide leak out from the blood [41–44]. In the present study, heavy inflammation in the form of macrophages and eosinophils was observed in the tetrathyridium migration tracks at 48 hours post-infection (Fig 3A and 3B). However, the number of inflammatory sites and severity of the inflammation in the intestinal wall decreased significantly by 96 hours post-infection (Figs 3C–3E and S2A–S2D). These results suggest that the inflammation induced by tetrathyridium penetration was dissipating as early as 96 hours post-infection.

The ES products released from some cestode species reportedly have immunomodulatory properties [14]. The ES products derived from *S*. *erinaceieuropaei* plerocercoids suppress the production of IL-1β and tumor necrosis factor-α inflammatory cytokines in macrophages stimulated with bacterial LPS [17–20]. The products from *M*. *vogae* have also been investigated and reported to suppress the production of inflammatory cytokines by bone marrow-derived dendritic cells stimulated by LPS [22, 23]. Therefore, we hypothesized that inflammatory cytokines at the injured tissue are down-regulated as an explanation for the early convergence of inflammation in the intestinal wall penetrated by tetrathyridium. Hence, we measured the gene expression levels of multiple genes associated with inflammation using exhaustive PrimerArray analysis. Consequently, most of the genes were confirmed not to be up-regulated consistently at 48 h post-infection although the tissue destruction and heavy inflammation were observed in the period in the histopathology (Figs 3 and S2E–S2G). In order to investigate the phenomenon, the expression levels of two representative inflammatory genes in acute infectious phase, IL-1β and CCL11, were evaluated properly with a time course by quantitative RT-PCR analysis. The expression levels of IL-1β and CCL11 in the infection group were lower than those of the non-infected group at 48 hours post-infection, suggesting that these genes were strongly suppressed by tetrathyridium infection (Fig 4G and 4H). IL-1β, a primary cytokine, induces inflammation in response to physical injury to cells as well as that caused by pathogens [28, 29], whereas CCL11 recruits eosinophils to combat parasitic helminths [33–34]. Strong suppression of cytokines might contribute to the early elimination of inflammation in the injured intestinal tissues and this is thought to avoid the parasite killing its host via exacerbation of the inflammation. However, present experiments provide no direct evidence that links the coincidences of an early healing of intestinal injuries and changed mRNA profiles in the intestine via oral *M*. *vogae* infection. Therefore, further study approaching a causative link between the two coincident phenomena is required.

In the present study, contrary to the low expression levels of cytokine-associated gene expression, 10 genes were up-regulated in all the tetrathyridium infection in the PrimerArray analysis. Five of these genes were cell growth factor associated genes (e.g., epidermal growth factor and activin receptor genes) (Fig 4D). This suggests that expression of the cell growth factor genes was up-regulated by tissue injury and blood vessel disruption after tetrathyridium migration, and that gene expression was kept at a higher level, thereby enabling tissue recovery in the host. The gene expression level of interleukin 17 (IL-17) receptors was also up-regulated in all the tetrathyridium infected mice (Fig 4D). IL-17 is an inflammatory cytokine, and intracellular signaling by it does not require signaling molecules like MyD88 and IL-1 receptor-associated kinase 4, which are essential for IL-1β signaling, while another molecule, ACT1, functions as an adapter for IL-17 receptors [45]. This suggests that the suppression of IL-1β expression by tetrathyridium might be caused by depression of these signaling molecules. The expression level of monocyte driven cytokines, CCR2, is down-regulated in all examined mice (Fig 4D). CCR2 is known to recruits monocyte to the intestine and promotes the accumulation of activated macrophage as a response infection with gastrointestinal parasitic helminth [46]. Therefore, the suppression of the gene expression could also contribute the disappearance of inflammatory responses via reduction the accumulation of macrophage in the tetrathyridium penetrated intestine. In the PrimerArray analysis, the expression levels of many inflammation response-encoding genes, including IL-1β and CCL11, varied between the two repeated experiments, whereas the variation within the three mouse individuals in the second experiment is small (Fig 4A–4C). The expression levels of these genes may fluctuate over a short period of time, as for IL-1β and CCL11 confirmed. As shown in Figs 1 and S1, the timing of tetrathyridium penetration through the intestinal wall slightly varies between independent experiments also shown in tracking observation of tetrathyridium migration (Figs 1B, 1C and S1A and S1B). The discrepancies in the expression levels of these genes between the two replicate experiments might be due to a slight shift in the timing of tetrathyridium penetration between the two experiments.

Collectively, our findings have shown that *M*. *vogae* tetrathyridium can strongly down-regulate the host’s inflammatory response in the injured intestinal wall, and this regulation might make the inflammatory loci caused by tetrathyridium migration disappear almost immediately. The death or illness of the host during the migration period, prior to proliferation of tetrathyridium in the peritoneal cavity, must be a disadvantage for parasite survival. Hence, our findings imply that effective migration without symptomatic enteritis accompanying the suppression of inflammatory responses in the host is an important survival strategy for *M*. *vogae*.

## Supporting information

S1 Fig Repeated experiment of tetrathyridium migration dynamics in mice. Result of another set of experiments. (A) The number of tetrathyridia on the surface of the liver, sticking to the small intestinal wall, or remaining in the abdominal cavity 24 and 48 hours after the oral infection. Squares, circles and triangles represent the small intestine, the abdominal cavity, and the liver, respectively. Error bars: standard deviation. (n = 3). (B) The number of tetrathyridia sticking to the small intestinal wall in each 1 cm area from the pylorus at 24 and 48 hours after the oral infection started. Intestinal areas 11cm from the pylorus are combined. White and black bars represent the number of tetrathyridia counted at 24 and 48 hour after the oral infection started, respectively. Error bars: standard deviation. (n = 3).
(JPG)

S2 Fig Examples of inflammatory responses, and repeated experiment of disappearance of the inflammatory responses. (A-D) Examples of HE stained histology corresponding inflammatory scores in tetrathyridia penetrated intestine; (A) score 1: longitudinal muscle layer, (B) score 2: submucosa and circular muscle layer, (C) score 3: submucosa, circular layer and longitudinal layer, and (D) score 4: lamina propria, submucosa, circular layer and longitudinal layer were damaged and infiltrated with inflammatory cells. Scale bars: 100 μm. (E-G) Examples of HE stained histology from a time course of inflammation intensity; Tissue of 48 h after oral injection of PBS (E), 600 tetrathyridia (F), and that of 96 h after oral infection of 600 tetrathyridia (G). The arrowheads represent inflammatory areas, scale bars: 200 μm. (H-J) Result of another set of experiments. Number (H) and total score (I) of inflammatory sites in the mouse intestinal wall at 48 and 96 hours after oral infection. Error bars: standard deviation, *p<0.05. (n = 3). (J) The scores distributions of each inflammatory sites in the mouse intestinal tissues. White and black dots represent each individual at 48 and 96 h post infection groups, respectively. (n = 3).
(JPG)

S1 Table Relative expression levels of 88 cytokine and cytokine-receptor genes and 8 housekeeping genes. (XLSX)
