# Supplementary figures and images for: Suppression of inflammatory genes expression in the injured host intestinal wall during Mesocestoides vogae tetrathyridium larvae migration
Source: PLoS Negl Trop Dis. 2020 Oct 13;14(10):e0008685. doi: 10.1371/journal.pntd.0008685 (PMC7598923; doi:10.1371/journal.pntd.0008685)

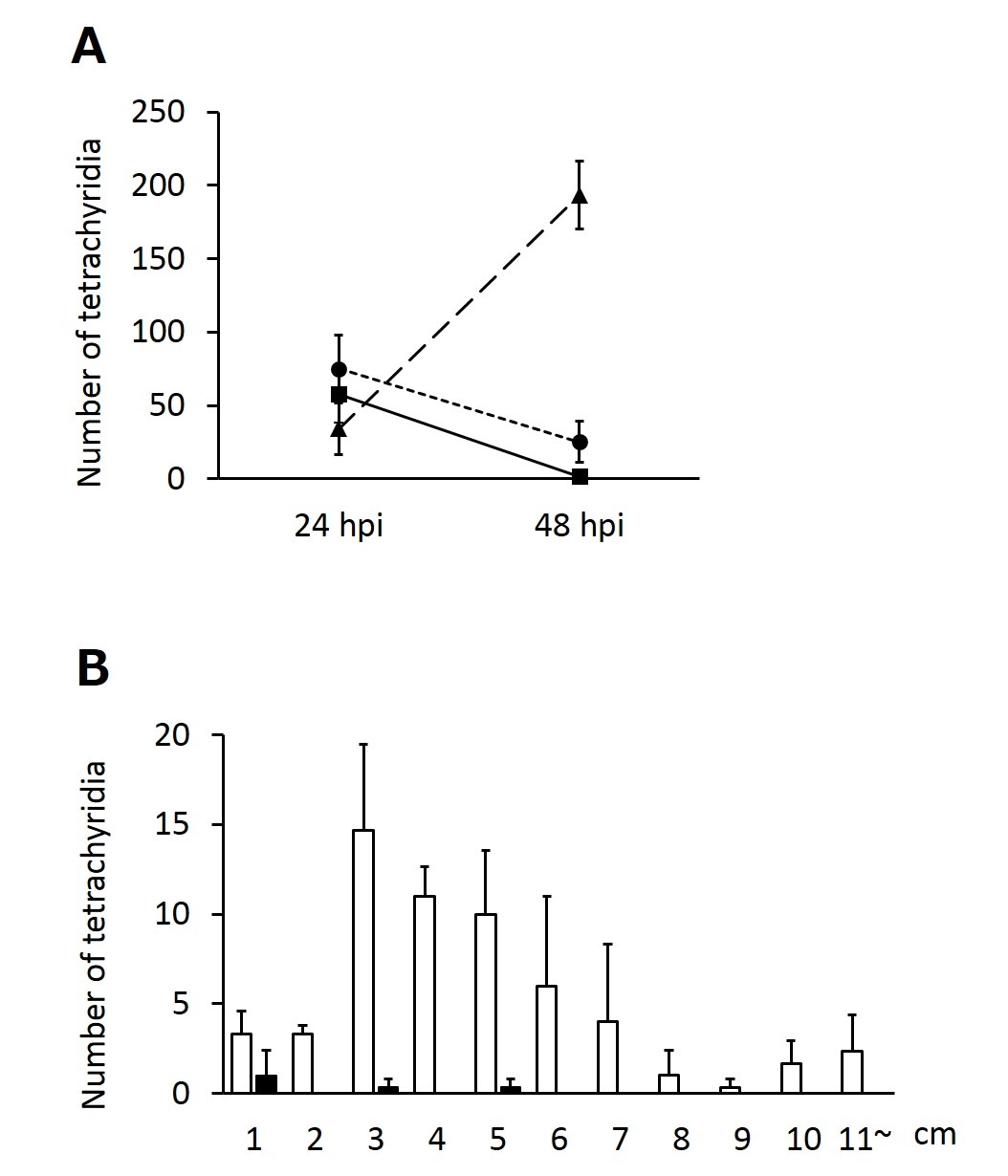

Supplement: S1 Fig — Result of another set of experiments. (A) The number of tetrathyridia on the surface of the liver, sticking to the small intestinal wall, or remaining in the abdominal cavity 24 and 48 hours after the oral infection. Squares, circles and triangles represent the small intestine, the abdominal cavity, and the liver, respectively. Error bars: standard deviation. (n = 3). (B) The number of tetrathyridia sticking to the small intestinal wall in each 1 cm area from the pylorus at 24 and 48 hours after the oral infection started. Intestinal areas 11cm from the pylorus are combined. White and black bars represent the number of tetrathyridia counted at 24 and 48 hour after the oral infection started, respectively. Error bars: standard deviation. (n = 3). (JPG) [file pntd.0008685.s001.jpg]

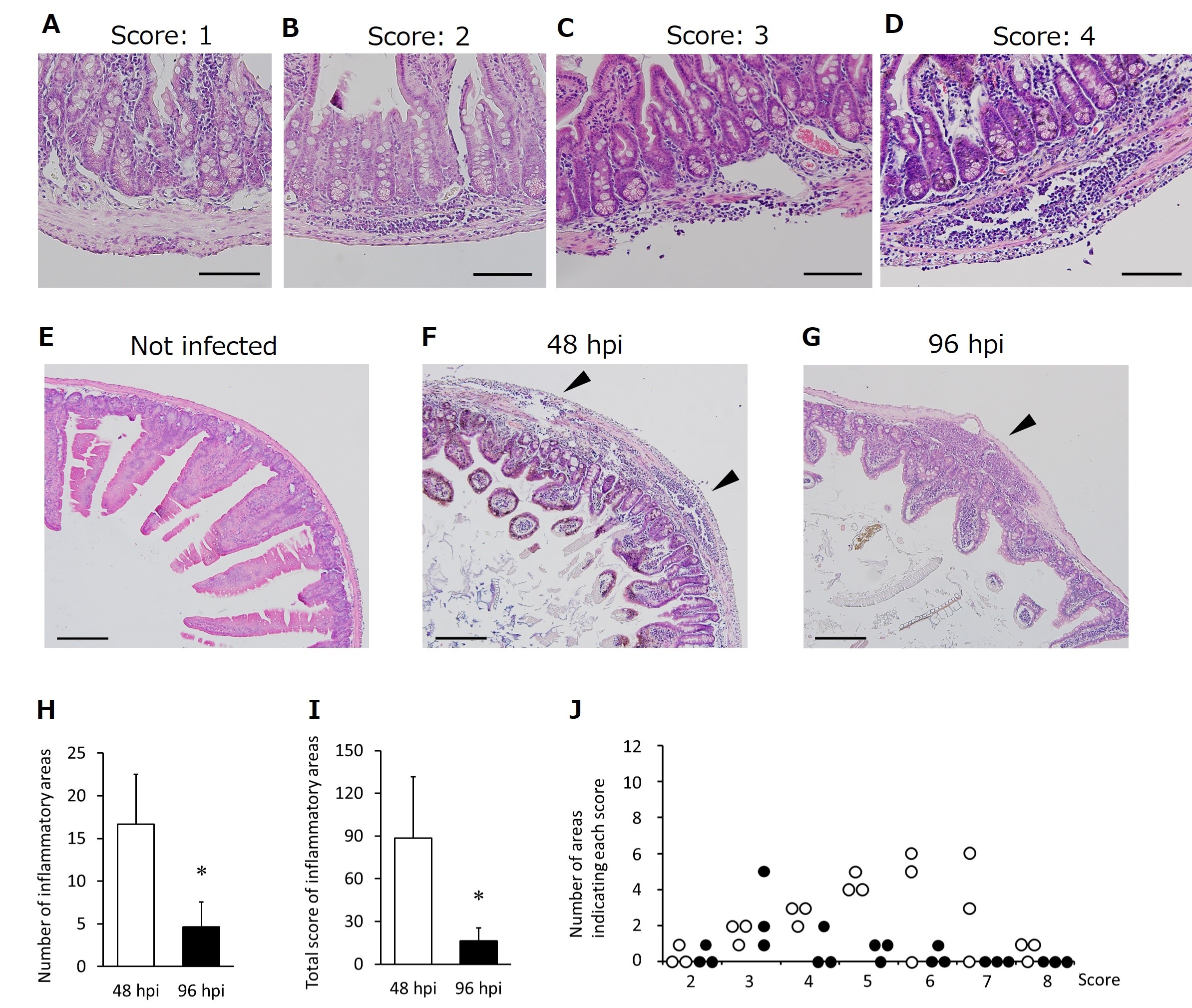

Supplement: S2 Fig — (A-D) Examples of HE stained histology corresponding inflammatory scores in tetrathyridia penetrated intestine; (A) score 1: longitudinal muscle layer, (B) score 2: submucosa and circular muscle layer, (C) score 3: submucosa, circular layer and longitudinal layer, and (D) score 4: lamina propria, submucosa, circular layer and longitudinal layer were damaged and infiltrated with inflammatory cells. Scale bars: 100 μm. (E-G) Examples of HE stained histology from a time course of inflammation intensity; Tissue of 48 h after oral injection of PBS (E), 600 tetrathyridia (F), and that of 96 h after oral infection of 600 tetrathyridia (G). The arrowheads represent inflammatory areas, scale bars: 200 μm. (H-J) Result of another set of experiments. Number (H) and total score (I) of inflammatory sites in the mouse intestinal wall at 48 and 96 hours after oral infection. Error bars: standard deviation, *p<0.05. (n = 3). (J) The scores distributions of each inflammatory sites in the mouse intestinal tissues. White and black dots represent each individual at 48 and 96 h post infection groups, respectively. (n = 3). (JPG) [file pntd.0008685.s002.jpg]
